# Perspectives of Multidisciplinary Professional Teams during Assessment Processes for ATD Selection in the Japanese Public Provision System

**DOI:** 10.3390/ijerph18052697

**Published:** 2021-03-08

**Authors:** Jun Suzurikawa, Yuki Sawada, Miwa Sakiyama, Motoi Suwa, Takenobu Inoue, Tomoko Kondo

**Affiliations:** 1 Department of Assistive Technology, Research Institute, National Rehabilitation Center for Persons with Disabilities, 4-1 Namiki, Tokorozawa-shi, Saitama 359-8555, Japan; saki381022@yahoo.co.jp (M.S.); inoue-takenobu@rehab.go.jp (T.I.); 2 Department of Occupational Therapy, Faculty of Medical Sciences, Teikyo University of Science, 2525 Yatsusawa, Uenohara-shi, Yananashi 409-0193, Japan; y-sawada@ntu.ac.jp; 3 Research Institute, National Rehabilitation Center for Persons with Disabilities, 4-1 Namiki, Tokorozawa-shi, Saitama 359-8555, Japan; suwa_motoi@yahoo.co.jp; 4 Department of Occupational Therapy, Faculty of Health Sciences, Kyorin University, 5-4-1 Shimorenjaku, Mitaka-shi, Tokyo 181-8612, Japan; tkondomtm@ks.kyorin-u.ac.jp

**Keywords:** assistive technology, rehabilitation services, social services, multidisciplinary collaboration, tacit knowledge

## Abstract

Selection of assistive technology devices (ATDs), which are imperative for persons with disabilities to improve their quality of life, requires collaboration of users and multidisciplinary professionals. However, it is still unknown how to design and implement an adequate collaborative work flow and a professional team. Under Japanese governmental ATD provision system, based on the application by clients, ATDs are mainly selected through collaborative processes with the clients and health professionals in public organizations, rehabilitation counseling centers (RCCs). By employing qualitative study methods in this study, we investigated the ATD selection process in which health professionals in RCCs collaboratively assess clients with physical disabilities so as to support them in selecting the adequate ATDs. To identify the perspectives required for ATD selection completely, the assessment processes were recorded and analyzed with a pseudo setting in two RCCs. Content analysis of the conversations between the client and professionals revealed the characteristics of the information exchanged in the assessment processes. A total of 760 assessment items were identified, thus indicating a broad array of interest. Despite the richness of information collected for the assessment, half of the assessment items did not have corresponding items in the documents that were employed during the prescription process. Thematic analysis of the interviews that followed revealed the common values and collaborative processes in ATD selection, which were shared and elaborated among the staff in daily social interactions. To facilitate implementation of ATD provision in various areas with few resources, it may be effective to convert this tacit-to-tacit knowledge sharing into a more explicit sharing by promoting analyses of good practices.

## 1. Introduction

An assistive technology device (ATD) is imperative for individuals with disabilities so as to improve their quality of life (QOL). The correct use of ATDs that are employed to replace impairments of body functions enhances activities of daily living, and sometimes subsequently facilitates their social participation in local communities [1]. In a number of high-income countries, the cost of ATDs is compensated for or reimbursed substantially by the public because the use thereof is indispensable for those with disabilities regardless of their personal economic status [2]. Therefore, it is important to ensure the effect of ATDs, and thus, maximize the cost/benefit efficiency.

However, ATDs are not always fully utilized by those who have acquired them. While ATDs include any devices, systems and products that optimize the functioning of individuals with disabilities, some require careful selection and/or adjustment. Unlike some universal-design products, for example, it is necessary to select such ATDs after careful assessment of the user’s body functions, daily activities, social participation, environments, and personal preferences. Inappropriate selection of ATDs has been known to lead to minor improvements in QOL and, sometimes, even to discontinued use or abandonment [3,4]. Depending on the user’s functional abilities, an ATD often needs to be adjusted or modified before use. Such assessment and adjustment require highly specialized knowledge in various fields related to medicine, rehabilitation, welfare system, and products, which users do not normally possess. Gitlin and Burgh [5] noted that some of such knowledge has a tacit and experience-based aspect that is difficult to learn. Consequently, without professional support comprising multidomain knowledge mismatches between users and ATDs may occur.

To prevent such mismatches, public provision systems usually include pre-provision processes in which various professionals are engaged for the assessment of users and selection of ATDs. This multidomain process is organized and administered in various ways in accordance with the local policies. The Global Cooperation on Assistive Technology initiative of the World Health Organization (WHO) proposed a 5-P framework comprising five interlinked domains, namely, people, policy, products, provision and personnel so as to enhance affordable access to ATDs globally [6]. This framework posits that policy should play an inclusive role to facilitate positive interactions among the rest of the domains. With the right process well-designed by policy, a user (people) is supported in selecting an ATD (product), that matches their needs, by rehabilitation professionals (personnel). 

Many studies have revealed that this pre-provision process has a significant influence on the extent to which provided ATDs will be used and contribute to improvement of QOL. Phillips and Zhao [7] in a survey in the United States identified four factors related to the abandonment of ATDs: “lack of consideration of user opinion in selection, easy device procurement, poor device performance, and change in user needs or priorities”. It clearly shows the importance of user involvement that the selection process of ATDs had a larger impact on their effects than the performance. Federici and Borci’s [8] survey in Italy also revealed various factors related to professional intervention that leads to ATD abandonment. A survey in Germany indicated that case managers may play an important role in the provision process [9]. These studies commonly indicate that the right design of the pre-provision processes during which professionals and users collaboratively reach decisions on the selection of ATDs is a critical factor in the maximization of the effect of ATDs usage.

The study conceptualized ATD provision processes as the Assistive Technology Assessment (ATA) process model [10], which includes the Matching Person and Technology (MPT) model [11]. Scherer’s MPT model emphasizes a user-driven process of ATD selection, where a user collaborates with multidisciplinary professionals. In the ATA process, the workflow for the ATD delivery service is similarly modeled to that of users. Before the ATD delivery, the service provider collects and evaluates user information, and collaborates with the user, to propose possible solutions that match the user’s needs.

Despite the importance of the pre-provision process, only a few reports have detailed how practitioners support users in selecting appropriate ATDs. Most studies on the processes of ATDs provision have investigated retrospectively the relationship between the effect of ATDs and the way the ATD was provided [3,12,13]. Consequently, the obtained insights have focused on prerequisites and requirements for enhanced provision processes and not on how these were achieved. While various studies have indicated that user involvement in the pre-provision process increases satisfaction with and the outcome of ATDs, other studies have examined the cooperative engagement of multidisciplinary professionals to realize an in-depth understanding of users’ needs. Although these insights are of high rationality, it remains unclear how such factors were installed and achieved in practice. Consequently, to clarify the proper process of ATDs provision, it is necessary to conduct more descriptive studies on processes in practice. 

To realize an enhanced understanding of the practice involved in the proper selection of ATDs, the purpose of this study was to investigate and characterize the assessment processes in the Japanese ATD provision system. In Japan, rehabilitation counseling centers (RCCs) play a central role in the provision of relatively expensive ATDs for adults with physical disabilities. RCCs are public organizations established by all the prefectures and the cities designated by government ordinance because of their large populations for counseling related to medical and vocational rehabilitation. ATDs provided through RCCs include power wheelchairs (PWCs), seating systems, prostheses and so on. RCCs are legally obligated to assign multidomain health and care professionals, including medical doctors, nurses, physical and occupational therapists, and social workers. Based on the application of ATD provision by clients, RCC staff assess functional abilities to validate whether the application meets the criteria for public provision. Then, staff members provide support in selecting ATDs that match clients’ needs. Compared with the processes of the ATA model, the pre-provision process in RCCs includes the majority of factors for ATD delivery services except for long-term follow-up and user support. A multidisciplinary team in an RCC has extensive experience of the pre-provision process; thereby, fundamental factors for effective practice may be inherent during the assessment and selection processes thereof.

## 2. Methods

We conducted a qualitative study, where a client presented a pseudo case of provision application of a PWC to two RCCs. The study encompassed two different types of investigations: first, content analysis of the pre-provision processes that were conducted by multidisciplinary teams in the RCCs, and second, thematic analysis of the following interviews to the RCC staff. The actions and conversations of staff members and a client were recorded in the pseudo setting. With the content analysis of the recorded prescription processes, we quantitatively characterized the patterns in which the multidisciplinary staff assessed the client. Interviews with the RCC staff supportively enhanced the understanding of the implicit values and intentions of the staff, as well as the tacit process of collaborative work.

### 2.1. Settings

Two RCCs, referred to as A and B, from metropolitan areas were selected for the investigation. The multidisciplinary teams of health professionals in both the RCCs were considered to have enough knowledge and skills for the pre-provision prescription processes of ATDs because they had been receiving a substantial number of applications. While RCC A team included a medical doctor (MD), social worker (SW), and rehabilitation engineer (RE), RCC B team comprised MD, SW, physical therapist (PT), and nurse as well as a provider of PWCs.

Because of the issues of confidentiality, it was difficult to observe real pre-provision processes in RCCs. Then we newly created the following pseudo situation for this study. A person with a disability, who had been appointed as a research cooperator for this study, applied for a new ATD. Subsequently, the multidisciplinary team performed the pre-provision assessment. This research cooperator was a male in his forties with complete chronic cervical spinal cord injury for 15 years. He was paralyzed below the fourth vertebrae of the cervical cord and used an electric-motor-driven manual wheelchair. Although he was able to maneuver it by himself, he could not keep a seating position because he had pressure injuries in the sacrum area. In the study, he was the client who applied for the provision of a PWC with powered tilting and reclining functions. Before the investigation, he agreed with the procedure of the investigation and his research cooperation.

### 2.2. Data Collection

The assessment process in the pseudo setting was performed once at each RCC. The staff members were informed of and agreed with the investigation in the pseudo setting in advance. In order to observe standard work flows, they were instructed to perform the usual assessment activities. The assessment processes including actions, interactions and conversations among the staff members and with the client were all video recorded by the authors. The short interactions among the staff members without the client were also recorded. The voice data in the video record were transcribed verbatim. The documents filled in during the assessment processes were also collected for analysis. 

Due to time constraints, the investigation was conducted within a day (Jan. and Feb. in 2012, for RCC A and B, respectively). Consequently, the authors collected information, which was normally requested by the RCCs, including application forms from the client, and had sent the information to the staff in the RCCs in advance. At the request of RRC B, the authors also visited the client’s residence and video recorded his home environment. This video data was presented to the staff of RCC B at the beginning of the investigation. Because some phases of the usual processes in the RCCs would be omitted from the investigation because of time constraints, we confirmed the whole phases in the following interviews.

### 2.3. Interview

Approximately two weeks after the pseudo assessment process, semistructured interviews were conducted with the staff members of both RCCs. The objectives of the interviews were first to identify differences between what the staff did in the pseudo setting and that conducted in the routine assessment of clients, and secondly to understand the perspectives involved in good practice in ATD provision. The core interview questions were as follows:

- In comparison to what you usually do, what was different in the pseudo setting?
- Was the information provided in advance sufficient?
- How have you learned knowledge and skills that are required for selection of ATDs?
- What are the requisites for reaching an appropriate decision in the pre-provision process?

Through those questions, they reflected on and enhanced the regular processes of their daily activities.

All the members of RCC A were interviewed simultaneously as a group (duration of the interview: 140 min.). While the SW and PT of RCC B were interviewed as a group (140 min.), the MD was interviewed on another day (90 min.) because of work shifts. The interviews were recorded and transcribed verbatim.

### 2.4. Analysis

To identify the patterns in the assessment processes, content analysis was conducted on the video-recorded prescription processes [14]. The conversations among the staff members and between the staff and the client were classified to afford a descriptive and quantitative understanding of the practice involved in ATD provision [14]. First, the conversations were coded based on the categories of International Classification of Functioning, Disability and Health (ICF) [15]. The data in the collected documents were also extracted and coded. The data that did not fit in the ICF categories were further coded by the unity of meaning. After all the codes were aggregated to reduce an overlap, assessment items were generated as minimum units of information that were collected during the assessment processes. Finally, the assessment items were classified into several groups that represented the major perspectives for assessment. To characterize the various aspects of the assessment processes, the assessment items were also scrutinized and classified in relation to the following perspectives: first, the temporal aspects—whether the items represented the client’s past, present, or future statuses; second, the communication flow—whether the items were transmitted from the client to the RCC staff, or vice versa; and third, the documentation status—whether the items had the corresponding entry columns in the collected documents.

Thematic analysis was complementarily employed to reveal the values and intentions of the professionals embedded in the processes of ATD provision and to enhance an understanding of the practice patterns, which were acquired through content analysis of the pseudo assessment process [14]. Because the quantitative results by the content analysis above were sometimes difficult to interpret without any qualitative cues, thematic analysis of the interviews was adopted to understand the practices in the RCCs better. After the verbatim transcripts were read several times, the meaning units were generated, and then collated into potential themes. From these themes, we extracted and named those relevant to the assessment processes unobserved in the pseudo settings and the intentions and perspectives that the professionals had. The named themes were reviewed and refined by all the authors, whose backgrounds included rehabilitation medicine, occupational therapy, rehabilitation engineering, and qualitative and quantitative research methods.

## 3. Results

This section presents the results obtained by content analysis of the pseudo assessment processes recorded in the RCCs and thematic analysis of the subsequent interviews to the staff members. The first four subsections display quantitative aspects of the assessment processes revealed by content analysis, indicating the richness of the perspectives that the multidisciplinary professionals had. The rest of the section describes the assessment phases not included in the pseudo setting and the intentions shared among the professionals during ATD selection with clients, which were extracted by thematic analysis of the interviews. 

### 3.1. Process Structures for ATD Selection

**Figure 1:**
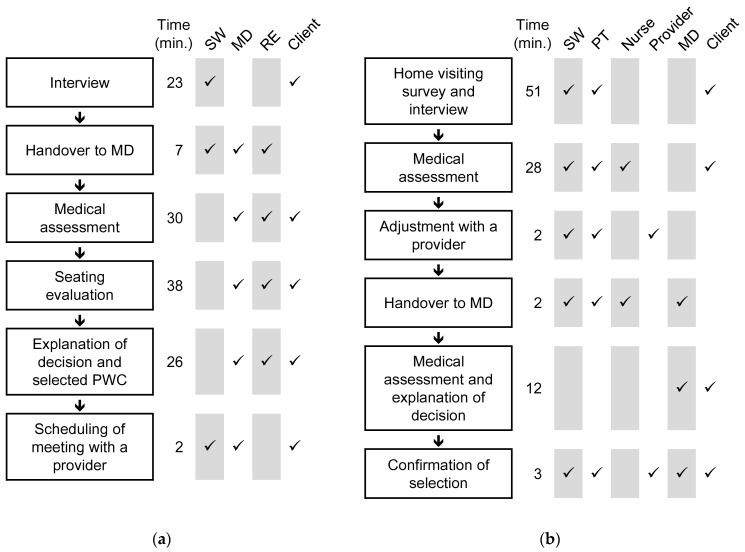
Recorded processes in the pseudo setting. The phase descriptions, the duration thereof, and participants for RCC A (**a**) and B (**b**) are depicted.

In Figure 1, the process flows recorded in the pseudo assessment processes in the two RCCs are depicted. RCC A and B required 126 and 125 min to draw up a prescription, respectively. The SWs in both RCCs played the primary role in initially collecting the client’s information. That information was subsequently transferred to MDs before they assessed the client directly. The MDs conveyed the decision on the provision of PWC to the client. 

The procedures to evaluate body structures and functions and environmental factors showed differences between the two RCCs. RCC A focused on evaluating the client’s seating condition, whereas RCC B expended more time on collecting information that covered the client’s general and various functioning. RCC B’s survey on residence and living environments also increased the range of information they collected. While the MD contributed to evaluation principally in RCC A, the allied health professionals were the primary contributors in RCC B. 

The documentation formats employed for assessment included Reception/Record of Application, Basic Information for Provision of PWC, Record of Physical Evaluation, Record of Seating Evaluation, Assessment of PWC Maneuver, Assessment of Mobility, Description of Medical Decision, and Prescription Sheet. The MDs and the nurse also reported information by employing free writing sheets.

### 3.2. Identified and Quantified Assessment Items for ATD Selection

As shown in Table 1, the analysis of the pseudo assessment recorded in RCC A and B identified 760 items in which the client and PWC to be provided were described. RCC A and B shared 114 of the 760 items, and had 267 and 379 specific items, respectively. Through the repetitive process of summarizing and comparing, two main categories and nine subcategories were generated. These are presented in the left column of Table 1.

One of the main categories, understanding for ATD selection, included the following seven subcategories: basic information, clarification of needs, information of the PWC in use, functional abilities, environmental factors, fitting evaluation for seating, and assessment of benefits and risks. Of these subcategories, functional abilities comprised 290 items, which was more than one-third of all the items. Fitting evaluation for seating also had a substantial number of items. However, the majority of the items were extracted from the process in RCC A because the seating evaluation including pressure distribution measurement was conducted only by them. In RCC B, environmental factors consisted of a large number of the items because they conducted the home visiting survey. Assessment of benefits and risks included aspects of how the client could increase participation by using the new PWC. 

Results of decision, which included 55 items, was another primary category. This category revealed RCCs’ decisions described within the staff members and to the client. One subcategory, judgment of provision, involved a description of the items to be provided that were defined in the provision system. The other, contents of ATD selection, included parts and structural specifications of the prescribed PWC. The decisions on the provision items were common in both RCCs, namely, a joystick-controlled power wheelchair with powered tilt-in-place and reclining functions. However, because there were differences in the assessment of the client, RCC A and B shared only an item in common in contents of ATD selection. RCC A focused on prescribing seating parts in detail, whereas RCC B only described standard wheelchair structures and left seating adjustment to the provider.

### 3.3. Temporal Aspects of the Assessment

The analysis of the pseudo assessment elucidated that the RCC staff attempted not only to understand the current conditions of the client but also to grasp the life course of the client. In Table 2, the items in the seven subcategories in understanding for ATD selection were reclassified into three categories that described the temporal aspect of the assessment, namely, past, present, and future. The items in the subcategories of basic information, information of the PWC in use, functional abilities, and environmental factors were mostly classified into present. In contrast, all the items in fitting evaluation for seating and assessment of benefits and risks were classified as future. The items in clarification of needs were classified into all three categories, while the largest number of items were classified into future.

The items classified into future were related to the changes required for or induced by a new PWC that was to be provided. The anticipation of such changes was based on the understanding of the client’s past and present situations. For example, the future items in environmental factors described the necessity of home modification to use the new PWC in a more enhanced manner, essential people who would arrange the modification, and changes of the way to use the living space. These considerations were supported by the substantial numbers of past and present items that were collected by means of the home visiting survey. The main focus of the future items in clarification of needs involved understanding how the client would change his lifestyle with a new PWC. To assess this change, the RCC staff determined his past and present needs, in other words, what had been and was achieved by the PWC in use.

### 3.4. Communication and Documentation during the Assessment Process

Communication between the client and the RCC staff and among the RCC staff was quantified by classifying the items in understanding for ATD selection into three new categories, namely, input, output, and documented, as shown in Table 3. Input and output represent the flow directions of information exchange. While the former involved the RCC staff collecting information from the client, the latter entailed the staff providing the client with information such as an explanation of the provision system, and confirmation of their recognition of the client’s situations. Documented means that the items were included in the documents used in at least one of the two RCCs. This category implies that among the RCC staff, the corresponding information was recognized explicitly as a requisite for assessment. 

Of the 705 items, 617 (87.5%) and 406 (57.6%) were classified into input and output, respectively. In the subcategory basic information, information of the PWC in use, functional abilities, and environmental factors, the input-to-total ratios were approximately 100%. This implies that the client mentioned the corresponding information directly. In contrast, these ratios were relatively low in clarification of needs, fitting evaluation for seating, and assessment of benefits and risks. This suggests that the RCC staff collected some of the information by observation. The high output-to-total ratio indicates that the RCC staff expended considerable effort to communicate with the client and avoid any misunderstandings. Even in highly specialized evaluations of body functions and seating, the RCC staff explained what they had assessed to the client as well as collected the relevant information. The number of the output items exceeded that of the input items in clarification of needs and assessment of benefits and risks. This suggests that the more needs for and benefits of PWC were not directly clarified by the client but anticipated by the staff, which was based on other assessments as well as their pre-existing knowledge and experiences as professionals. These were subsequently confirmed with the client. 

The overall ratio of the documented items was 46.5%. While the individual ratios in the subcategories ranged from 0% to 63%, that in basic information reached 100%. The majorities of the items in fitting evaluation for seating and assessment of benefits and risks were not written in the documents. More than the half of the items were included in the documents used in clarification of needs and functional abilities. This quantification implies that the RCC staff implicitly collaborated with one another to collect enough information for ATD selection rather than just sharing explicit listing of the assessment items that had to be gathered. The documented assessment items appeared to cover only a core range required, which was expanded by collecting undocumented items on demand.

### 3.5. Usual Assessment Processes

**Figure 2:**
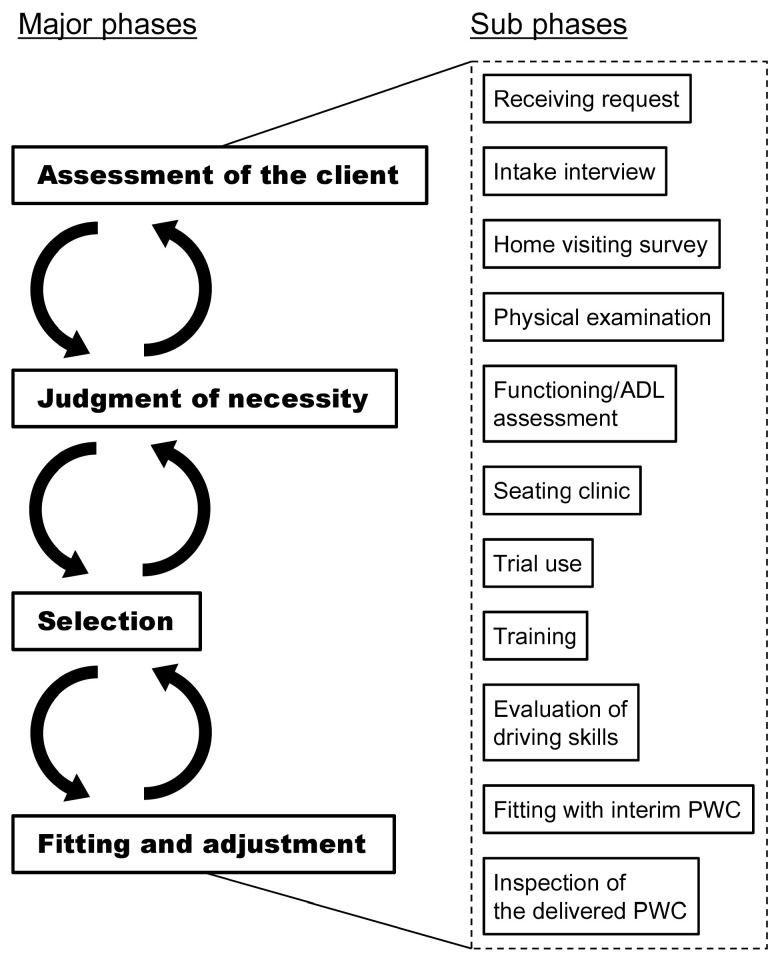
The ATD provision process derived from the interview. Four major phases and sub-phases are depicted.

In relation to the difference between the pseudo and a usual setting, the unobserved processes that were usually involved in ATD selection were revealed by the analysis of the interviews. As depicted in Figure 2, in usual processes, four major phases related to ATD provision were identified, assessment, judgment of necessity, selection of ATDs, and fitting and adjustment. The major phases encompassed the subphases, which are also illustrated in Figure 2. The boundaries of the phases were unclear and some phases were repeated if necessary. The subphases that constituted the major flow also influenced one another seamlessly. Therefore, it was difficult to identify the clear links between the major and sub phases. While some subphases occurred in both RCCs, others only took place in one. The occurrences seemed to be subject to the restriction of time, availability of staff members and equipment, and the clients’ conditions. 

Considering the phases of ATD provision, which were identified in the interviews, what was observed in the pseudo setting was a mere part of the whole provision process. In particular, the latter phases for selecting and adjusting PWC were omitted from the investigation. The role of the RCCs was not limited to ATD selection but included confirmation of fitting with the delivered PWC. It should be noted that the assessment items that have been identified do not include those considered in the latter phases.

### 3.6. Intentions for Good Practice

Three themes were found from the analysis of the interviews to the RCC staff. 

The first was finding an essential solution to raise the quality of client’s life. This intention did not focus only on finding the best ATD among other ATDs, but placed emphasis on broadening the scope to select the essential means among all possibilities including improvement of physical function, house modification, and use of home health service. The MD of RCC B expressed his intention when he meets clients as the following:

“Despite the client specifying what he wants, that may not be what he really wants. We would like to know what kind of life he wants to have, what he likes, what his dream is, and what he really cares…”

This encompassing scope is evident in vast number of the assessment items in Table 1 as well as the repetitive mention of functional abilities and clarification of needs. 

The second theme was supporting clients’ life, including their future. The staff members intended to support their clients’ entire life by asking them how they could improve their lives by using ATDs. The intension appeared in what the SW of RCC A said: “I try to look ahead at the hope of clients and what they want to do in their lives.” The MD of RCC A explained his holistic view of clients: “There are many kinds of aspects of the future, such as physical aspects, social aspects and so on. I try to see all these aspects of the future and compound them to (select the optimal ATD).” If the clients were not able to imagine their lives with ATDs or were unaware of the possibilities thereof, the RCC staff endeavored to consult and educate them. This intention is in accordance with the fact that the communication between the client and staff members was not only prevalent in the present situation, but also in the past and future (Table 2). Furthermore, there were more output items in clarification of needs and assessment of benefits and risks than input items (Table 3). 

The third theme that appeared as a shared intention was provision as public expense. The MD of RCC B said:

“In case, even if a client requested a PWC with a much higher function, such as a stand-up structure, it cannot be provided here. In such a case, if we just say ‘No, it cannot be according to our criteria.’, the client only feels unsatisfied. … So it is most difficult (important) to reach an agreement, that is, the client gets to understand our judgement.”

RCCs play the role of judging if the requested ATDs were right for public expenses, and the staff members consistently exercise fairness throughout the processes. Their sense of fairness is not only exhibited by simply following the regulations but also in identifying facts in cases where the selected ATDs are considered indispensable for clients. The MD of RCC B also described, “I am not trying to limit the provision by virtue of the regulations, rather I consider if the ATD is absolutely necessary for the person’s social participation and quality of life.” This intention was not evident in the pseudo setting, but clearly manifested during the interviews.

Associated with those shared intentions, each professional had his/her own unique focus during the assessment process. For example, the MDs focused on body structures, physical prognosis, integrating information, and making a final decision. The SWs focused on assuring provisional standards, explaining the provisional system to clients, conveying the clients’ wishes, and understanding the clients’ daily situations. PT focused on clients’ body structures and physical and daily functions, assessing clients’ physical environments, and selecting appropriate ATDs. RE focused on fitting body and ATDs and arranging the best products by selecting such from the market or manufacturing these themselves.

### 3.7. Team Building for Collaborative Work

The theme of collaborative work was relaying the information and ideas. In both the RCCs, there were only a few short meetings in which all the staff members participated and exchanged the information in relation to the pseudo assessment processes. Furthermore, in the interviews, they also shared that great deal of information usually remained unwritten even though some paperwork such as prescription documents was inevitable. Despite these factors hampering the sharing of information, the RCC staff perceived that they could communicate with one another sufficiently enough to collect assessment information collaboratively. The RCC staff related that they could exchange and gather assessment information through the questions that other professionals asked to the clients as if they were working in the relay team. As depicted in Figure 1, many of the phases in the pseudo assessment processes involved more than two different professionals, and thus, the staff members who had participated in the prior phase relayed information to members who were not present. Through relaying, complete and accurate information was gathered.

The RCC staff acknowledged team building approaches to establish such smooth communication and information sharing. Because the ATD selection processes were complicated and dependent on experience-based individual skills with high specialty, manualization of ease could suffer from a lack of contextual and implicit factors to adequately adjust the range of information to be collected according to clients’ situations. Rather, the enhancement of mutual understanding among the professional members facilitated the collaborative collection of assessment information, although indirectly, more efficiently. The RCC staff also related the importance of the active exchange of opinions among the members in occasions such as case conferences. Such reflections could reveal the overlooked items during the assessment and afford a chance to know other professionals’ specialty.

## 4. Discussion

### 4.1. Multidisciplinary Perspectives in the Preprovision Processes

The observation of the pre-provision processes in Japanese RCCs revealed that the staff members collaboratively and multilaterally assessed clients for ATD selection. The assessment information collected during the processes included a wide range of factors related to the physical, social, and personal situations of the client. The RCC staff also paid much attention to how the living status, needs, and social participation of the client would change in the future with a new ATD. Because the expense of providing an ATD is a public cost, it is imperative that decisions are fair. However, the time available for ATD selection were limited despite the richness of assessments. These features of the pre-provision processes in the RCCs required a well-established team of multidisciplinary professionals. 

Previous studies on ATD provision have revealed that multidisciplinary collaboration is a key factor for increasing the effects received by using them [10,16,17,18]. Because the needs and contexts for ATDs largely vary according to clients even with the same diagnosis and symptoms, social and environmental factors play an important role [19]. Multidisciplinary teams of health professionals can provide a comprehensive understanding of clients, and thus, match ATDs with the clients. The results in this study implied that the two RCCs that were examined implemented such a multidisciplinary approach adequately. The staff members with different specialties successively assessed the client and shared the information implicitly. The assessment items identified by the content analysis were qualitatively and quantitatively rich, and covered a wide range of the client’s aspects (Table 1). This abundant feature achieved by the collaborative work of the multidisciplinary team is consistent with the literature. 

The appropriate ATD was carefully and collaboratively selected with the client and the RCC staff; however, the follow-up process, which is recognized as critical for successful ATD use, was not highlighted either in the pseudo setting or the interviews. RCCs in Japan are organizations designed for temporal consultation and do not offer a long-term rehabilitation service, which is mainly a part of the medical service within local communities. However, to optimize ATD on a long-term basis, the development of systems for continuous follow-up is necessary. Moreover, active and cross-sectional collaboration beyond the RCC staff, such as health professionals of health care facilities in local communities and those in municipal governments is a potential solution.

### 4.2. Client-Centered Selection of ATDs

Client centeredness during the selection processes of ATDs is another fundamental factor that influences effectiveness of acquired ATDs. A number of quantitative studies have confirmed the importance of the client-centered approach [7,8,13]. User involvement and participation in the selection processes were proven to enhance the outcomes of ATDs while the lack of these factors can be a predictor of abandonment. Consequently, health professionals who are in charge of ATD selection are needed to carefully consider clients’ opinions including the goals to be achieved with a new ATD and inform them of the decisions adequately [12,20,21].

In the pseudo setting of this study, the RCC staff intensively informed the client of their recognitions and assumptions that were derived from the assessment results. As shown in Table 3, the analysis on the flow directions of information during the assessment revealed that more than half of the total assessment items were conveyed to the client. This observation reveals that the RCC staff intentionally facilitated client involvement in the ATD selection process. Martin et al. [12] showed that clients’ feeling “well informed” has a positive impact on satisfaction with the use of acquired ATDs. Riemer-Reiss and Wacker [22] also pointed out a significant inverse relationship between consumer involvement and disuse of ATDs. Thus, the active information sharing observed between the RCC staff members and the client is effective to ensure continuous use of the provided PWC and to decrease the possibility of abandonment.

A characteristic factor found in ATD selection in RCCs was the public nature of ATD provision thereof. While clients have the right to obtain appropriate ATDs, the range and criteria of public provision should be simultaneously considered. The RCC staff declare their concerns about judging the properness of provision and share it with the client. However, in the pseudo setting, the balance between the criteria and the client’s request was not observed when there was a conflict between the two factors. Further investigation is necessary to reveal and evaluate a practicable solution for these conflicts.

The investigation in this study shed light on another aspect of client-centeredness. As revealed in the interview, the RCC staff perceived the necessity not only to assess but also to sometimes guide the client. Because ATD users do not have specialized knowledge of all the factors that have to be considered for appropriate device selection, they can overlook some possibilities, in other words, what can be achieved with new devices. On such occasions, it is the role of professionals to provide the users with the right information and insights [23]. Actually, the results displayed in Table 3 indicate that the RCC staff presented various needs that the client had not mentioned. It is highly probable that their recommendations and suggestions were based on careful observations and interpretations of the client. This kind of guidance will be beneficial for clients to expand their possible goals with ATDs and subsequently make a better choice. Future research should focus on confirming post-provision outcomes resulting from such collaborative goal settings through surveys on the ATD users who experienced the ATD selection process in RCCs.

### 4.3. Implications for the Implementation of ATD Provision

The tacit way for the RCC staff to assess the client was elucidated quantitatively by the content analysis of the pseudo assessment processes. As shown in Table 3, less than half of the total assessment items were included in the documents that were used in the RCCs. However, the subsequent interview with the RCC staff revealed that the staff members communicated with one another adequately and shared collected information during the pseudo assessment processes. They also related being involved in an intentional team-building to realize collaborative work. Although a long-term effort is likely to be inevitable in order to establish an efficient team of multidisciplinary professionals, excess formalization or manualization of the ATD selection process was denied outright. They perceived that it was difficult to cope flexibly with clients’ various situations in a fixed way of assessment.

In relation to the knowledge management framework proposed by Nonaka [24], the form of team building examined in the RCCs includes aspects of “socialization”, which means tacit-to-tacit knowledge transfer through the shared experiences in daily social interactions [25]. Consequently, the documented assessment items can be considered as the result of “externalization”, which implies tacit-to-explicit knowledge translation. This translation, however, seemed to be incomplete and thus, was unable to fully cover the assessment procedures in practice. The RCC staff still depended predominantly on the tacit knowledge shared among the professional members.

The presence of tacit knowledge has been widely debated in the field of rehabilitation [26,27,28]. Clinical assessment and decision making require substantial experience-based knowledge and are difficult to convert into explicit knowledge. Therefore, the manner in which experienced professionals care or assess clients cannot be easily learned by those with less experience. On the other hand, endeavors to analyze and formalize tacit practices, which were clinically recognized to be effective, have been reported [29,30,31]. Such knowledge management approaches have the potential to help practitioners to improve the quality of service according to experience-based knowledge that was clinically accumulated over a long period. However, it should still be noted that balancing explicit and tacit knowledge is necessary because of the limits to the advantages of standardizing and quantifying clinical practices [32].

Practices in ATD selection are also known to include experience-based tacit knowledge [5]. In the literature, a number of efforts have already been made to partly formalize or standardize the assessment processes in ATD selection [33,34,35]. These systematic knowledge management approaches were intended to facilitate the spread of appropriate ATD provision systems. The complex nature of ATD selection highlighted in this study also emphasizes the necessity for the further translation of experience-based practices in well-established ATD provision organizations. Because of the shortage of trained professionals, it may be difficult to build a multidisciplinary team for ATD selection, especially involving tacit-to-tacit knowledge transfer. Such situations are likely to be prevalent in low- and middle-income countries as well as in the rural areas of high-income countries. To overcome this problem, the externalization of tacit knowledge is a practical solution for the fast implementation of an ATD provision system.

### 4.4. Limitations of the Study

As the purpose of this study was to realize an in-depth observation and understanding of the practices in public ATD provision in Japan, the following limitations should be noted before the findings are generalized.

First, the two RCCs that were investigated were among more than 70 such organizations in Japan. Furthermore, both were located in urban areas. Therefore, both were likely to receive more applications for ATDs than the average RCC in Japan. It is assumed that this had a positive influence on the results of this study because the staff members had sufficient experience through daily practices. However, organization-specific approaches, biases, and omissions in the pre-provision processes may have been prevalent to cope with many clients. Consequent misunderstandings due to the small sample numbers could not be avoided.

Second, the study can include biases due to cultural differences among countries. Characteristics of the implementation of ATD provision in Japan may influence the results of the investigation, and the consequent conclusions. This possibility should be considered when deploying information obtained in this setting and comparing it with that in other countries.

Third, unlike other studies that have considered the outcomes of the provided ATDs, neither evaluation of the appropriateness of the pre-provision processes investigated nor interviews to real clients were conducted in this study. As noted previously, the analysis results of the pseudo assessment suggest that the recorded processes in the two RCCs involved multidisciplinary collaboration and user centeredness, which were revealed to be imperative for adequate ATD selection in the literature. Despite these observed features, it should be noted that the processes and the decisions did not completely guarantee effective outcomes. 

Fourth, the use of a pseudo setting may affect the results. RCC staff members were informed of the investigation; hence, they could potentially change their behaviors from their normal practices. Results of the content analysis may include such artifacts that are difficult to tell apart.

Finally, the pseudo setting in this study focused only on the provision of a PWC for a client with spinal cord injury. Difference in ATD categories and the severity of disabilities may result in different assessment approaches [19]. Therefore, the results in this study possibly lack aspects that have to be considered for other ATDs and disabilities.

## 5. Conclusions

Overall, this study clarified how ATDs were selected based on careful assessment of the clients in Japanese RCCs, both quantitatively and qualitatively. Content analysis of the conversations recorded during the pre-provision processes revealed the richness of the information collected for the assessment of the client: the total number of the assessment items reached 760. Thematic analysis of the interviews to the RCC staff after the investigation enhanced understanding of the practice. The analysis results highlighted the user-centered approach by the multidisciplinary professionals. The RCC staff made endeavors to communicate with the client and to inform the client of their recognitions and assumptions that were derived from the assessment results. The staff members had intention even to consult and educate clients when they did not notice new possibilities with ATDs. The results provide evidence of the active role which multidisciplinary professionals play to support individuals with disabilities in ATD selection. This perspective prompts ATD provision systems to adopt the pre-provision phase wherein users and health professionals collaboratively examine and select a new ATD.

Another feature identified in the pre-provision processes was the tacit way of assessing the client. The half of the assessment items did not have corresponding items in the documents that were employed in the organizations. The staff members, however, perceived that they could collaboratively collect sufficient information to make decision on the ATD selection. Team building through the daily interaction among the staff occurred to be the factor that enabled sharing of the implicit experience-based knowledge for ATD selection. Because this tacit-to-tacit knowledge transfer requires a long-term effort and sufficient human resources, tacit knowledge externalization is a practical solution for the fast implementation of an ATD provision system. Even with a well-systematized work flow, it is still important for policy makers to facilitate nurturing active teams that enable tacit-to-tacit knowledge sharing.

## Figures and Tables

**Table 1:** Identified perspective categories and number of assessment items.

Categories	Number of Assessment Items					
	A and B	A	Only A	B	Only B	Total
**Understanding for ATD selection**						
Basic information	23	38	15	34	11	49
Clarification of needs	10	24	14	52	42	66
Information of the PWC in use	6	18	12	29	23	41
Functional abilities	49	94	45	245	196	290
Environmental factors	18	24	6	88	70	94
Fitting evaluation for seating	2	143	141	17	15	158
Assessment of benefits and risks	0	1	1	6	6	7
**Results of decision**						
Judgment of provision	5	7	2	7	2	9
Contents of ATD selection	1	32	31	15	14	46
Total	114	381	267	493	379	760

**Table 2:** Temporal aspects of the assessment.

Categories	Number of Assessment Items (%)						
	Total	Past		Present		Future	
Basic information	49	11	(22.4)	38	(77.6)	0	(0)
Clarification of needs	66	13	(19.7)	12	(18.2)	41	(62.1)
Information of the PWC in use	41	3	(7.3)	37	(90.2)	1	(2.4)
Functional abilities	290	2	(0.6)	286	(98.6)	2	(0.6)
Environmental factors	94	5	(5.3)	79	(84.1)	10	(10.6)
Fitting evaluation for seating	158	0	(0)	0	(0)	158	(100)
Assessment of benefits and risks	7	0	(0)	0	(0)	7	(100)
Total	705	34	(4.8)	452	(64.1)	219	(31.1)

**Table 3:** Communicating directions and documentation statuses of the assessment items.

Categories	Number of Assessment Items (%)						
	Total	Input		Output		Documented	
Basic information	49	47	(95.9)	49	(100)	49	(100)
Clarification of needs	66	46	(69.7)	51	(77.3)	37	(56.1)
Information of the PWC in use	41	41	(100)	11	(26.8)	11	(26.8)
Functional abilities	290	284	(97.9)	187	(64.5)	183	(63.0)
Environmental factors	94	86	(91.5)	38	(40.4)	30	(31.9)
Fitting evaluation for seating	158	113	(71.5)	63	(39.9)	18	(11.4)
Assessment of benefits and risks	7	0	(0)	7	(100)	0	(0)
Total	705	617	(87.5)	406	(57.6)	328	(46.5)

## Data Availability

The data that support the findings of this study are available upon reasonable request to the corresponding author.

## References

[1] Østensjø S., Carlberg E.B., Vøllestad N.K. (2005). The use and impact of assistive devices and other environmental modifications on everyday activities and care in young children with cerebral palsy. Disabil. Rehabil..

[2] Lilja M., Mansson I., Jahlenius L., Sacco-Peterson M. (2003). Disability Policy in Sweden: Policies Concerning Assistive Technology and Home Modification Services. J. Disabil. Policy Stud..

[3] Federici S., Meloni F., Borsci S. (2016). The abandonment of assistive technology in Italy: A survey of National Health Service users. Eur. J. Phys. Rehabil. Med..

[4] Hocking C. (1999). Function or feelings: Factors in abandonment of assistive devices. Technol. Disabil..

[5] Gitlin L.N., Burgh D. (1995). Issuing Assistive Devices to Older Patients in Rehabilitation: An Exploratory Study. Am. J. Occup. Ther..

[6] MacLachlan M., Scherer M.J. (2018). Systems thinking for assistive technology: A commentary on the GREAT summit. Disabil. Rehabil. Assist. Technol..

[7] Phillips B., Zhao H. (1993). Predictors of Assistive Technology Abandonment. Assist. Technol..

[8] Federici S., Borsci S. (2016). Providing assistive technology in Italy: The perceived delivery process quality as affecting abandonment. Disabil. Rehabil. Assist. Technol..

[9] Henschke C. (2012). Provision and financing of assistive technology devices in Germany: A bureaucratic odyssey? The case of amyotrophic lateral sclerosis and Duchenne muscular dystrophy. Health Policy.

[10] Federici S., Scherer M.J., Borsci S. (2014). An ideal model of an assistive technology assessment and delivery process. Technol. Disabil..

[11] Scherer M.J., Craddock G. (2002). Matching Person & Technology (MPT) assessment process. Technol. Disabil..

[12] Martin J.K., Martin L.G., Stumbo N.J., Morrill J.H. (2011). The impact of consumer involvement on satisfaction with and use of assistive technology. Disabil. Rehabil. Assist. Technol..

[13] Borg J., Larsson S., Östergren P.-O., Rahman A.S.M.A., Bari N., Khan A.H.M.N. (2012). User involvement in service delivery predicts outcomes of assistive technology use: A cross-sectional study in Bangladesh. BMC Health Serv. Res..

[14] Vaismoradi M., Turunen H., Bondas T. (2013). Content analysis and thematic analysis: Implications for conducting a qualitative descriptive study. Nurs. Health Sci..

[15] Duggan C.H., Albright K.J., Lequerica A. (2008). Using the ICF to code and analyse women’s disability narratives. Disabil. Rehabil..

[16] Verza R., Carvalho M.L.L., Battaglia M.A., Uccelli M.M. (2006). An interdisciplinary approach to evaluating the need for assistive technology reduces equipment abandonment. Mult. Scler. J..

[17] Watson A.H., Ito M., Smith R.O., Andersen L.T. (2010). Effect of Assistive Technology in a Public School Setting. Am. J. Occup. Ther..

[18] Casey K.S. (2011). Creating an assistive technology clinic: The experience of the Johns Hopkins AT Clinic for patients with ALS. Neurorehabilitation.

[19] Eggers S.L., Myaskovsky L., Burkitt K.H., Tolerico M., Switzer G.E., Fine M.J., Boninger M.L. (2009). A Preliminary Model of Wheelchair Service Delivery. Arch. Phys. Med. Rehabil..

[20] Brandt Å., Hansen E.M., Christensen J.R. (2019). The effects of assistive technology service delivery processes and factors associated with positive outcomes—A systematic review. Disabil. Rehabil. Assist. Technol..

[21] Wielandt T., McKenna K., Tooth L., Strong J. (2006). Factors that predict the post-discharge use of recommended assistive technology (AT). Disabil. Rehabil. Assist. Technol..

[22] Riemer-reiss M.L., Wacker R.R. (2000). Factors Associated with Assistive Technology Discontinuance among Individuals with Disabilities. J. Rehabil..

[23] Steel E.J., Layton N.A., Foster M.M., Bennett S. (2016). Challenges of user-centred assistive technology provision in Australia: Shopping without a prescription. Disabil. Rehabil. Assist. Technol..

[24] Nonaka I. (1994). A Dynamic Theory of Organizational Knowledge Creation. Organ. Sci..

[25] Nonaka I., Toyama R. (2003). The knowledge-creating theory revisited: Knowledge creation as a synthesizing process. Knowl. Manag. Res. Pract..

[26] Mattingly C. (1991). What is Clinical Reasoning?. Am. J. Occup. Ther..

[27] Dijkers M.P., Murphy S.L., Krellman J. (2012). Evidence-Based Practice for Rehabilitation Professionals: Concepts and Controversies. Arch. Phys. Med. Rehabil..

[28] Sinclair E., Radford K., Grant M., Terry J. (2014). Developing stroke-specific vocational rehabilitation: A soft systems analysis of current service provision. Disabil. Rehabil..

[29] Panzarasa S., Maddè S., Quaglini S., Pistarini C., Stefanelli M. (2002). Evidence-based careflow management systems: The case of post-stroke rehabilitation. J. Biomed. Inform..

[30] Kontos P.C., Naglie G. (2009). Tacit knowledge of caring and embodied selfhood. Sociol. Health Illn..

[31] Bright F.A., Boland P., Rutherford S.J., Kayes N.M., McPherson K.M. (2012). Implementing a client-centred approach in rehabilitation: An autoethnography. Disabil. Rehabil..

[32] Greenhalgh J., Flynn R., Long A.F., Tyson S. (2008). Tacit and encoded knowledge in the use of standardised outcome measures in multidisciplinary team decision making: A case study of in-patient neurorehabilitation. Soc. Sci. Med..

[33] Fuhrer M.J., Jutai J.W., Scherer M.J., DeRuyter F. (2003). A framework for the conceptual modelling of assistive technology device outcomes. Disabil. Rehabil..

[34] Scherer M.J., Sax C., Vanbiervliet A., Cushman L.A., Scherer J.V. (2005). Predictors of assistive technology use: The importance of personal and psychosocial factors. Disabil. Rehabil..

[35] Smith E.M., Gowran R.J., Mannan H., Donnelly B., Alvarez L., Bell D., Contepomi S., Ennion L., Hoogerwerf E.-J., Howe T. (2018). Enabling appropriate personnel skill-mix for progressive realization of equitable access to assistive technology. Disabil. Rehabil. Assist. Technol..

